# Editorial: Graphical and imaging technology in thoracic surgery: a game changer

**DOI:** 10.3389/fsurg.2023.1208574

**Published:** 2023-05-09

**Authors:** Jiaxi He, Eugenio Pompeo

**Affiliations:** ^1^ Department of Thoracic Surgery, The First Affiliated Hospital of Guangzhou Medical University, Guangzhou, China; ^2^ Guangzhou Institute of Respiratory Disease & China State Key Laboratory of Respiratory Disease, Guangzhou, China; ^3^ Department of Thoracic Surgery, Policlinico Tor Vergata University, Rome, Italy

**Keywords:** thoracic surgery, VATS, image technology, three dimension (3D), bronchoschopy

**Editorial on the Research Topic** Advances in graphical and Imaging technology in thoracic surgery

Significant technological advancements in thoracic surgery have been witnessed over the years, which also changes the entire “game,” from detection and diagnosis to surgical treatment, revolutionizing the clinical practices of thoracic surgeons. These game changers elevate the accuracy of diagnosis, increase the preciseness of surgical procedures, reduce complications, and improve patient outcomes. In this issue, several reports discuss the advances in graphical and imaging technology in thoracic surgery and what they have changed.

Computed Tomography (CT) is the most widely used imaging technology in thoracic surgery. Over these years, high-resolution images, broader window width and level, and three-dimensional reconstruction have been well-developed, revealing previously undetected details and enabling functional evaluation (1). Despite the structural information of the organs and tissue, vascular structures and blood supply could be demonstrated with the contrast agents. Especially in the sublobar resection, identifying the locations of fissures, segmental planes, tracheobronchial and vascular are crucial.

In this issue, Li et al. reported a study exploring the correlation between uncommon right posterior segmental bronchus and recurrent artery with the help of three-dimensional CT bronchography and angiography (3D-CTBA). It was a retrospective study with 600 patients with pulmonary nodules who underwent segmentectomy. The anatomical variations of bronchus and arteries were delicately displayed, showing that patients with defective and splitting B2 had a higher incidence of recurrent artery crossing the intersegmental plane. However, those without abnormal B2 still had a 10% possibility of crossing recurrent arteries. Regarding the anatomical abnormalities, Lyu et al. reported a case of a tri-lobed left lung and a literature review of the accessory lobe variants. The patient with a small pulmonary nodule in the left accessory lobe was misdiagnosed in the left lower lobe. Because of this anatomical variation, surgeons should relocate the nodule and change their surgical plan during the operation. The review indicated the incidence of accessory fissures in the left lung was 13.5%, and the accessory lobe in the left lung was 2%. Most of these cases were identified in autopsy and operation. The identified rate of accessory lobes in chest x-ray and traditional CT ranged only from 7.3% to 32%. Thus, a pre-operational evaluation with high-resolution CT images and delicate 3D reconstruction considerably lowers operational risk and reduces misdiagnosis.

Quantitative CT could be used in lung function evaluation based on morphological features. Wang et al. performed a meta-analysis investigating the correlation between CT parameters and airflow obstruction in chronic obstructive pulmonary disease (COPD) patients. They noticed that lung attenuation percentage, density, airway wall area proportion, and air trapping index were more closely correlated with expiratory CT features than inspiratory, and these could be applied to evaluate the severity of COPD. With recent advances in imaging technology, both morphological characteristics and even function parameters can be obtained from CT scans for comprehensive evaluation (2).

Bronchoscopy developments also account for an essential part of thoracic surgery in pulmonary nodule localization and treatment (3). As shown in a case report from Yang et al. an esophageal foreign body was portioned by a laser beam and removed endoscopically. Removing long and punctured esophageal foreign bodies is risky and usually completed by thoracotomy. The report showed a safe, feasible, and alternative method to solve this problem, in which graphical technology advances played an inevitable role.

In the final report on this topic, Wang et al. repaired a tracheal defect with an autologous pericardial patch and a 3D custom-made carbon fiber stent after the malignant peripheral nerve sheath tumor resection. Any structural changes in the airway can lead to aerodynamic alterations in ventilation, inducing different clinical symptoms. Therefore, airway reconstruction is never easy. The authors reported that a delicate 3D model of the trachea and tumor was created with CT data. Then, the stent was prepared according to the model using 3D printing, which reconstructed the luminal structure avoiding floating of the patch and tracheal stenosis. The ideal outcome of the patient proved the innovativeness and feasibility of this approach regardless of the financial disadvantage.

The advances in graphical and imaging technology have greatly benefited every aspect of thoracic surgery. As a game changer, it elevates the entire levels of diagnosis, operation, and prognosis in thoracic surgery and is still on a fast track moving forward. VR and Meta technologies are bringing it to a higher level. This issue of Frontiers is the tip of the iceberg in this field. The applications of this technology are broad and becoming more comprehensive as time goes on.
